# The Mitochondrial Epigenome: An Unexplored Avenue to Explain Unexplained Myopathies?

**DOI:** 10.3390/ijms23042197

**Published:** 2022-02-16

**Authors:** Archibold Mposhi, Lin Liang, Kevin P. Mennega, Dilemin Yildiz, Crista Kampert, Ingrid H. Hof, Pytrick G. Jellema, Tom J. de Koning, Klaas Nico Faber, Marcel H. J. Ruiters, Klary E. Niezen-Koning, Marianne G. Rots

**Affiliations:** 1 Department of Pathology and Medical Biology, University Medical Center Groningen, University of Groningen, Hanzeplein 1, 9713 GZ Groningen, The Netherlands; mposhiarchibold@gmail.com (A.M.); l.liang@umcg.nl (L.L.); k.p.mennega@umcg.nl (K.P.M.); p.g.jellema@umcg.nl (P.G.J.); m.h.j.ruiters@umcg.nl (M.H.J.R.); 2 Department of Gastroenterology and Hepatology, University Medical Center Groningen, University of Groningen, Hanzeplein 1, 9713 GZ Groningen, The Netherlands; k.n.faber@umcg.nl; 3 Department of Laboratory Medicine, Laboratory of Metabolic Diseases, University Medical Center Groningen, University of Groningen, Hanzeplein 1, 9713 GZ Groningen, The Netherlands; dileminyildiz@gmail.com (D.Y.); crista.kampert@gmail.com (C.K.); i.h.hof@umcg.nl (I.H.H.); k.e.niezen-koning@umcg.nl (K.E.N.-K.); 4 Department of Genetics, University Medical Center Groningen, University of Groningen, Antonius Deusinglaan 1, 9713 AV Groningen, The Netherlands; t.j.de.koning@umcg.nl; 5 Department of Clinical Sciences, Lund University, Lasarettgatan 40, 221 85 Lund, Sweden

**Keywords:** mitochondrial epigenome, myopathies, mitochondrial DNA methylation, cytochrome B

## Abstract

Mutations in either mitochondrial DNA (mtDNA) or nuclear genes that encode mitochondrial proteins may lead to dysfunctional mitochondria, giving rise to mitochondrial diseases. Some mitochondrial myopathies, however, present without a known underlying cause. Interestingly, methylation of mtDNA has been associated with various clinical pathologies. The present study set out to assess whether mtDNA methylation could explain impaired mitochondrial function in patients diagnosed with myopathy without known underlying genetic mutations. Enhanced mtDNA methylation was indicated by pyrosequencing for muscle biopsies of 14 myopathy patients compared to four healthy controls, at selected cytosines in the Cytochrome B (*CYTB*) gene, but not within the displacement loop (*D-loop*) region. The mtDNA methylation patterns of the four healthy muscle biopsies were highly consistent and showed intriguing tissue-specific differences at particular cytosines with control skin fibroblasts cultured in vitro. Within individual myopathy patients, the overall mtDNA methylation pattern correlated well between muscle and skin fibroblasts. Despite this correlation, a pilot analysis of four myopathy and five healthy fibroblast samples did not reveal a disease-associated difference in mtDNA methylation. We did, however, detect increased expression of solute carrier family 25A26 (*SLC25A26*), encoding the importer of S-adenosylmethionine, together with enhanced mtDNA copy numbers in myopathy fibroblasts compared to healthy controls. To confirm that pyrosequencing indeed reflected DNA methylation and not bisulfite accessibility, mass spectrometry was employed. Although no myopathy-related differences in total amount of methylated cytosines were detected at this stage, a significant contribution of contaminating nuclear DNA (nDNA) was revealed, and steps to improve enrichment for mtDNA are reported. In conclusion, in this explorative study we show that analyzing the mitochondrial genome beyond its sequence opens novel avenues to identify potential molecular biomarkers assisting in the diagnosis of unexplained myopathies.

## 1. Introduction

Mitochondrial myopathies are a group of mitochondrial diseases that develop due to mitochondrial dysfunction in muscle tissue, which results in impaired muscle function and weakness [1]. Patients who develop mitochondrial myopathies have a perturbed energy metabolism because mitochondria are the hub for cellular energy production. Mitochondria contain their own (mtDNA), which encodes 13 core constituents of the mitochondrial respiratory complexes (I, III, IV, and V) and numerous noncoding RNA molecules [2,3]. Mitochondrial genes are either expressed from the heavy strand or the light strand of mtDNA, with promoters in the so-called *D-loop* [4,5]. Despite containing their own genetic material, mitochondria heavily depend on the expression of the nDNA, which encodes the majority of mitochondrial proteins, including those necessary for the electron transport chain (ETC), as well as for replication and transcription of the mitochondrial genome [2,6,7].

Mitochondrial diseases can be categorized in two groups: primary mitochondrial diseases, which occur due to inherited mutations in either mtDNA or nDNA in genes encoding ETC proteins; and secondary mitochondrial dysfunction, which describes diseases that can be acquired secondary to environmental effects [8]. In both subgroups, mutations in mtDNA or nDNA are associated with mitochondrial disease. For instance, a mutation in the mitochondrial NADH dehydrogenase subunit 1 (*ND1)* gene or a mutation in the nuclear NADH-ubiquinone oxidoreductase 75 kDa subunit 1 (*NDUFS1*) gene (both encoding subunits of the ETC complex I) are associated with muscle weakness, cardiomyopathy, and grey and white matter abnormalities [9,10]. In addition, mtDNA mutations in the cytochrome B (*CYTB*) gene (involved in ATP synthesis) predispose to mitochondrial myopathies [11,12]. However, there are patients who present all the clinical signs and symptoms of myopathy, but do not harbor mutations in either mtDNA or nDNA during clinical diagnostic screening for whole genome. In such cases of unexplained clinical presentation, it is difficult to diagnose or predict a patient’s predisposition to a mitochondrial disease. It is possible that epigenetic changes in the nDNA could be a contributing factor. Indeed, a study conducted on patients with congenital myopathies identified over 3500 differentially methylated nuclear genes and an increase in DNA methyltransferase 1 (*DNMT1*) expression [13]. In a study addressing skeletal muscle myopathy, mitochondrial dysfunction and nDNA hypermethylation were observed in mice with hyperhomocysteinemia [14]. In addition to nuclear DNA methylation, differential methylation of mtDNA has been proposed as a potential biomarker for disease [6,15].

Previous studies have shown that there is an association between mitochondrial dysfunction and mitochondrial gene expression differences [16,17]. However, it remains largely unknown how mtDNA gene expression is exactly regulated [5,18]. The mitochondrial copy number was considered to be the most important parameter [19,20], but differential mtDNA expression can occur without differences in the copy number [21]. Mechanistically, we reported that artificially induced mtDNA methylation affected mitochondrial gene expression and mitochondrial function [22]. Indeed, mtDNA expression profiles have been associated with differential levels of mtDNA methylation in liver [16] and cancer [23], and various studies indeed reported differential mtDNA methylation to be associated with tissue type [24] and disease [6,15].

Some studies attributed the existence of mtDNA methylation to technical artifacts, as the compact packaging of mtDNA would associate with a low accessibility of the DNA for, e.g., bisulfite conversion (often used to determine DNA methylation, as 5-methylcytosine (5mC) is protected against this chemical conversion) [25,26]. However, other studies disputed this [6,15] and linearization of the mtDNA is considered to circumvent this issue. Detailed whole-genome bisulfite sequencing (WGBS) data pointed out that mtDNA methylation occurred predominantly in a strand-specific, non-CpG manner potentially explaining some failures to demonstrated CpG methylation [27,28]. Using mass spectrometry, which avoids the technical problem of inaccessibility of mtDNA for bisulfite conversion, conflicting data also were reported regarding the existence of mtDNA methylation [27,29]. The introduction of long-read nanopore sequencing promises to settle this controversy, but again, technical (annotation) issues prevent straightforward conclusions with findings of differential CnonG methylation [30], differential CpG methylation [30,31], and absence of CpG methylation [32]. In support of mtDNA methylation, DNA methylation-modulating enzymes (DNMTs and ten-eleven translocation (TET) methylcytosine dioxygenases (TETs)) have been detected in mitochondria, and inactivation of DNA methyltransferases resulted in a decrease in mtDNA methylation [27,28].

In this study, we collected muscle biopsies and skin fibroblasts from patients who presented clinical signs and symptoms of myopathy, but who did not reveal known mutations in either the mtDNA or the nDNA after genetic screening. For these patients, we hypothesized that mtDNA methylation may be a factor leading to impaired mitochondrial function and the subsequent decline in ATP generation. The aim of this study thus was to investigate whether the observed decrease in ATP-generating capacity in muscle biopsies from “unexplained myopathy” patients may be explained by a change in mtDNA methylation or other mitochondrial epigenome parameters [18].

## 2. Results

### 2.1. High SAM Concentrations Induced mtDNA Methylation

**Figure 1:**
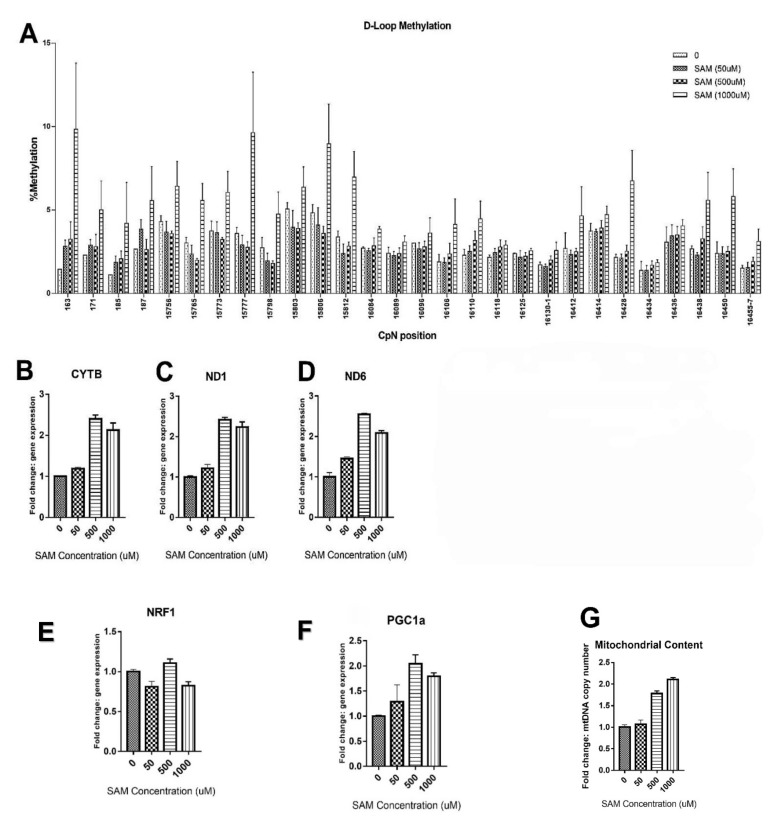
MtDNA pyrosequencing analysis and gene expression in HSkMCs treated with increasing concentrations of SAM (0–1000 µmol/L). (**A**) *D-loop* pyrosequencing (163–187, 16084–16457). Mitochondrial and nuclear gene expression: (**B**) *CYTB*; (**C**) *ND1*; (**D**) *ND6*; (**E**) *NRF1*; (**F**) *PPARGC1A* encoding PGC-1α; (**G**) mitochondrial copy number.

To set up the pyrosequencing readout for mtDNA methylation, we first explored whether treatment with S-adenosylmethionine (SAM) induced mtDNA methylation by treating human skeletal muscle cell line cells (HSkMCs) with increasing concentrations of SAM. Indeed, enhanced mtDNA methylation levels were detected for 16 out of 28 CpNs at the highest SAM concentration (Figure 1A). The average methylation percentage for all analyzed cytosines increased with increasing concentrations to 1.9-fold at 1000 µmol/L SAM compared to the untreated controls. Interestingly, SAM dose-dependently increased mitochondrial gene expression (*CYTB, ND1,* and *ND6*) in HSkMCs up to 2-fold at 1000 µmol/L (Figure 1B–D). Although SAM did not affect nuclear respiratory factor 1 (*NRF-1*) expression (Figure 1E), the expression of the nuclear gene *PPARGC1A* encoding peroxisome proliferator-activated receptor gamma coactivator 1 alpha (PGC-1α) was increased in SAM-treated HSkMCs (Figure 1F). This SAM-induced increase in *PPARGC1A* expression was accompanied by a 2-fold increase in mtDNA content (Figure 1G)**.**

### 2.2. CYTB mtDNA Methylation Was Higher in Muscle Biopsies from Myopathy Patients Compared to Healthy Controls

**Figure 2:**
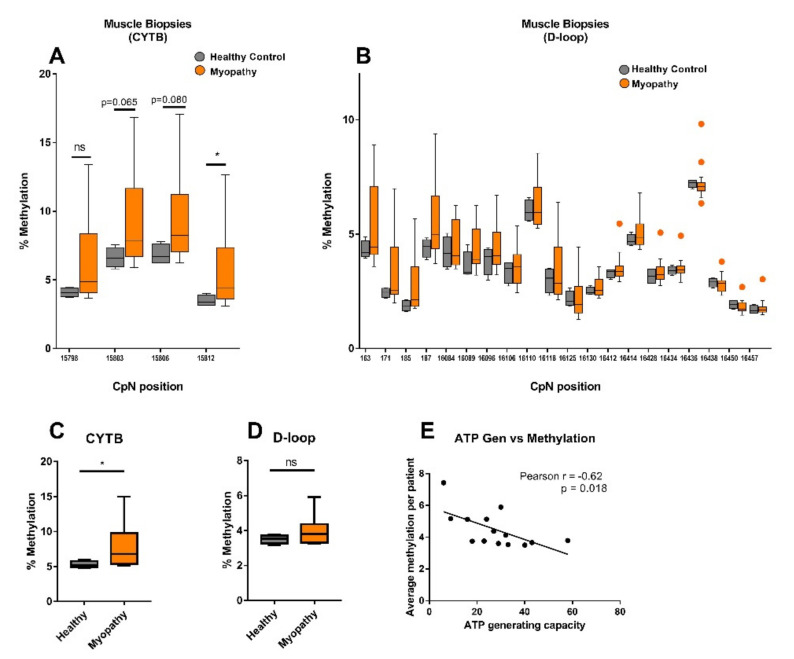
Increased mtDNA methylation in muscle biopsies from myopathy patients compared to healthy samples. MtDNA pyrosequencing analysis of muscle tissue from four healthy controls and 14 myopathy patients. Methylation was quantified by pyrosequencing in the (**A**) *CYTB* gene (15798–15812) and (**B**) *D-loop* region (163–187, 16084–16457). (**C**) The average methylation of all 4 CpNs in the *CYTB* region; (**D**) the average methylation of all 20 CpNs in the *D-loop* region; (**E**) correlation between mtDNA methylation versus ATP-generating capacity (ATP Gen) in muscle biopsies of myopathy patients (each dot represents one patient sample). Pearson’s correlation was used to calculate the correlation between average methylation per patient for all tested cytosines and ATP-generating capacity. Significance was demonstrated as * *p* ≤ 0.05, compared to the healthy controls, ns: no significant difference.

Subsequent pyrosequencing of muscle biopsies of four nonmyopathy (healthy) controls within *CYTB* (15798–15812) (Figure 2A) and the *D-loop* (163–187 and 16084–16457) (Figure 2B) showed a mtDNA methylation pattern with surprisingly little interdonor variation (methylation values ranging from 1.7% ± 0.2 to 7.3% ± 0.8). Methylation levels were typically higher for muscle biopsies from 14 myopathy patients at all four positions in the *CYTB* region, but reached statistical significance only for the CpG at position 15,812, when compared to the healthy controls (medians of 4.4% (95%CI, 3.6–7.4) versus 3.4% (95%CI, 3.2–3.9), respectively; *p* < 0.05; Figure 2A). Methylation levels of the 20 analyzed positions in the *D-loop* from myopathy patients were comparable to healthy muscle samples (Figure 2B).

The median of the average methylation percentages across all four CpN positions within the analyzed *CYTB* region was 1.3-fold higher for myopathy patients compared to healthy controls (6.8% versus 5.1%, respectively; *p* < 0.05) (Figure 2C), while there was no difference observed for the 20 positions within the *D-loop* (Figure 2D). The average mtDNA methylation levels of all the analyzed cytosines per myopathy patient sample indicated a negative correlation with the ATP-generating capacity of the corresponding muscle tissue (*r* = −0.62; *p* = 0.018) (Figure 2E), warranting more detailed research into mtDNA methylation and ATP levels.

### 2.3. Distinct mtDNA Methylation Patterns in Muscle Tissue Versus Skin Fibroblasts

**Figure 3:**
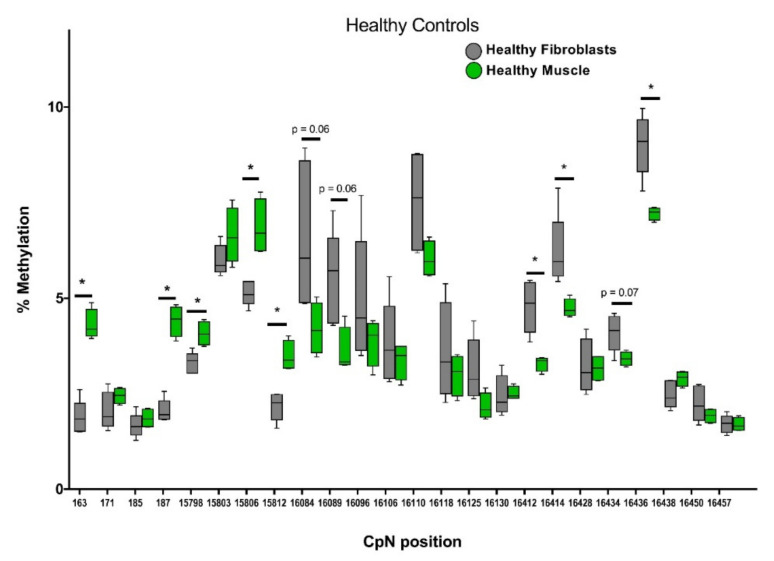
Tissue-specific mtDNA methylation patterns in muscle biopsies vs. healthy skin fibroblast cell lines from healthy individuals. Muscle tissue and skin fibroblasts were obtained from healthy (nonmyopathy) individuals (*n* = 4 and *n* = 5, respectively). *D-loop* (163–187 and 16084–16457) and *CYTB* (15798–15812) regions were pyrosequenced to quantify mtDNA methylation. Significance was demonstrated as * *p* ≤ 0.05 compared to the healthy fibroblasts.

To explore whether mtDNA methylation in skin fibroblasts could be used as proxy for muscle tissue, pyrosequencing was conducted on primary fibroblasts generated from five healthy skin biopsies and compared to four healthy muscle biopsies for our selected CpN positions within *CYTB* and the *D-loop* (Figure 3). At 8 out of the 24 cytosine positions, significant differences in methylation were observed between muscle tissue and cultured skin fibroblasts.

**Figure 4:**
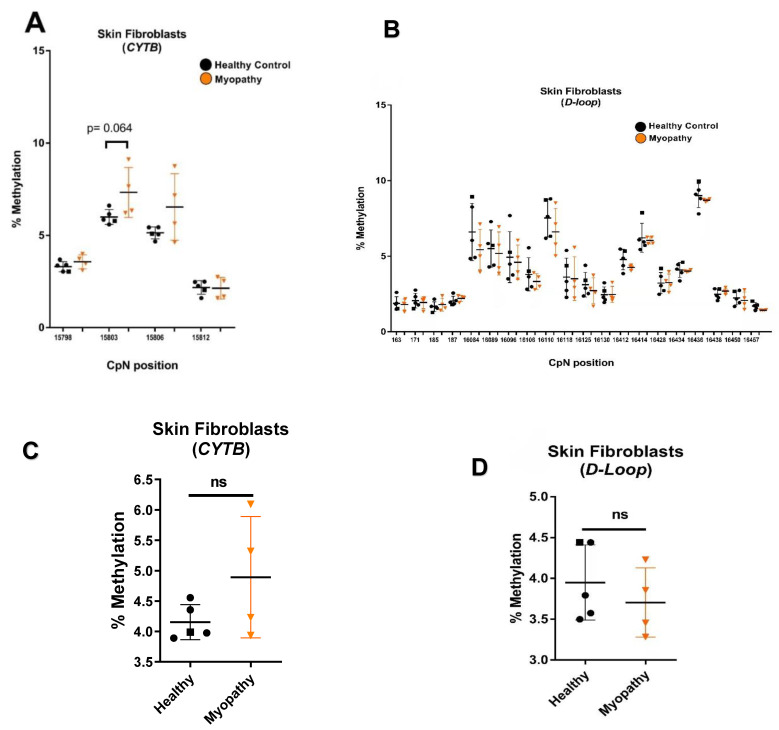
Higher *CYTB* methylation in skin fibroblasts from myopathy patients compared to healthy samples. MtDNA pyrosequencing analysis of skin fibroblasts from healthy controls and myopathy patients. Methylation was assessed for the (**A**) *CYTB* gene (15798–15812) (healthy controls, *n* = 5; myopathy patients, *n* = 4) and (**B**) *D-loop* region (163–187, 16084–16457) (healthy controls, *n* = 5; myopathy patients, *n* = 4). (**C**) The average methylation of 4 cytosines in the *CYTB* region (15798–15812); (**D**) the average methylation of all 20 cytosines in the *D-loop* region. (Healthy controls are represented as dots, HDFa16 represented as square and myopathy patients are represented as triangles). ns: no significant difference.

Next, we analyzed four myopathy skin fibroblast samples. Again, *CYTB* mtDNA methylation values were generally higher than those determined for the five control skin fibroblast samples (Figure 4A). Though the differences were not significant in the small sample size of four myopathy-tissue- and five healthy-tissue-derived skin fibroblasts, the CpT at position 15803 in the *CYTB* gene showed a borderline significant increase in methylation in myopathy-tissue-derived skin fibroblasts (*p* = 0.064; Figure 4A). To analyze the *CYTB* region in more detail, we designed additional primer pairs (14698–14760, 15375–15438, and 15699–15761), and again screened primary skin fibroblasts from three healthy donors and five myopathy patients for mtDNA methylation levels. Using the same patient material, highly reproducible cytosine methylation levels were observed, but no differences were detected between healthy- and myopathy-derived skin fibroblasts at any of the 21 additional analyzed positions in the *CYTB* gene (Figure S1). In addition, no differences in mtDNA methylation were detected in the 20 positions in the *D-loop* (Figure 4B). As a result of these observations, differences in mtDNA methylation also were not found between healthy-donor- and myopathy-patient-derived skin fibroblasts when averaging the levels of four analyzed cytosine positions in the *CYTB* gene (Figure 4C) or all the analyzed cytosines in the *D-loop* (Figure 4D).

**Figure 5:**
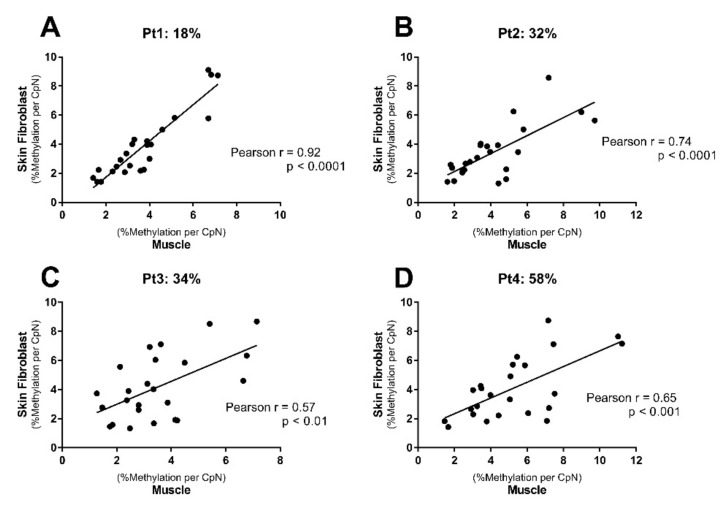
MtDNA methylation in muscle tissue correlated with mtDNA methylation in cultured skin fibroblasts of the same patient. Muscle tissue and skin fibroblasts were obtained from four myopathy patients, with ATP-generating capacity of (**A**) 18% (Pt1); (**B**) 32% (Pt2); (**C**) 34% (Pt3); and (**D**) 58% (Pt4), respectively. Pearson’s correlation analysis was applied using CpN positions within the *D-loop* (163–187, 16084–16457) and *CYTB* (15798–15812). (Each dot represents one CpN).

An exploratory correlation analysis was performed between mtDNA methylation levels in muscle tissue and the corresponding skin fibroblasts from four myopathy patients (Pt1, Pt2, Pt3, and Pt4, with ATP-generating capacities of 18%, 32%, 34%, and 58%, respectively) for the analyzed CpN positions within the *D-loop* and *CYTB* (15798–15812) (Figure 5). Interestingly, a correlation between CpN location-specific mtDNA methylation in muscle tissue and the corresponding skin fibroblasts was observed for all four patients. Notably, patient Pt1, with the lowest ATP-generating capacity (18%), showed the strongest and most significant correlation between muscle tissue and cultured skin fibroblasts (*r* = 0.92; *p* < 0.0001; Figure 5A). These results suggested that cultured skin fibroblasts show promise as a proxy for muscle tissue in myopathy diagnosis.

### 2.4. Mitochondrial Gene Expression Was Not Increased in Patient Skin Fibroblasts

**Figure 6:**
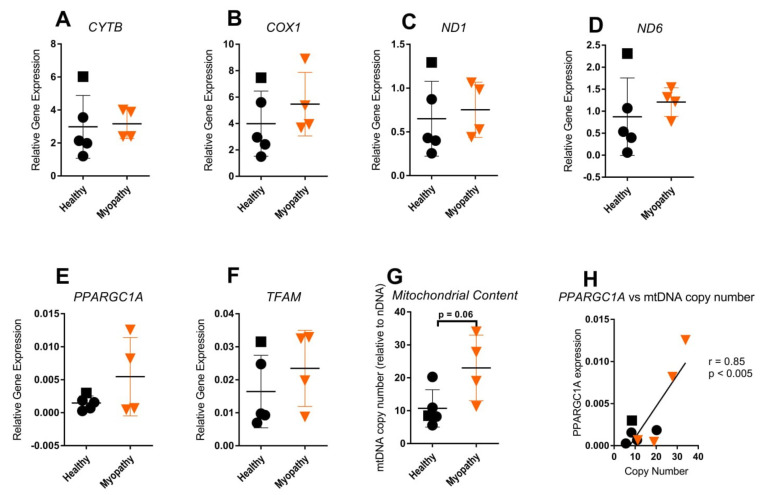
Mitochondrial and nuclear gene expression associated with mitochondrial biogenesis and mtDNA copy number in skin fibroblasts from myopathy patients compared to healthy controls: (**A**) *CYTB*; (**B**) *COX1*; (**C**) *ND1;* (**D**) *ND6*; (**E**) *PPARGC1A*; (**F**) *TFAM*. (**G**) Mitochondrial content (mtDNA copy number) and (**H**) correlation analysis for *PPARGC1A* expression vs. copy number (note that two samples are overlapping). Healthy controls (*n* = 4, represented as dots, and HDFa16 represented as square) and myopathy patients (*n* = 4, represented as triangles). None of the comparisons (**A**–**F**) reached statistical significance.

Next, we compared the expression of selected mtDNA-encoded genes (*CYTB, COX1, ND1,* and *ND6)* (Figure 6A–D) and nuclear-encoded genes involved in mitochondrial biogenesis (*PPARGC1A* and transcription factor A *(TFAM*)) (Figure 6E,F) in skin fibroblasts from healthy controls versus those from myopathy patients. No significant difference in the expression of these genes was observed. Still, two out of four fibroblast lines from myopathy patients showed a high expression of the nuclear-encoded *PPARGC1A* gene, as compared to the highest expression found in the five control fibroblast lines. Moreover, the median mtDNA copy number was more than doubled for myopathy-derived fibroblasts, though this only reached borderline significance when compared to healthy-tissue-derived fibroblasts (*p* = 0.060; Figure 6G). Most notably, the *PPARGC1A* expression showed a positive correlation with the mitochondrial copy number when taking both the control and myopathy fibroblasts into consideration (*r*= 0.85; *p* < 0.005; Figure 6H).

### 2.5. Gene Expression of Mitochondrial Transporters in Skin Fibroblasts

**Figure 7:**
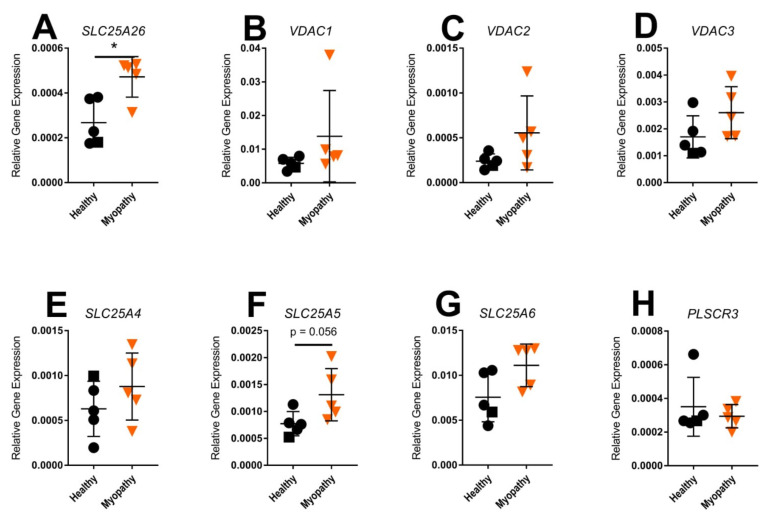
Expression of genes encoding mitochondrial transporters in skin fibroblasts of myopathy patients compared to healthy controls: (**A**) *SLC25A26*; (**B**–**D**) *VDAC1–3*; (**E**–**G**) *SLC25A4–6*; (**H**) *PLSCR3*. Healthy controls (*n* = 4, represented as dots, and HFDa16 represented as squares) and myopathy patients (*n* = 5, represented as triangles). Significance was demonstrated as * *p* ≤ 0.05 compared to the healthy controls.

In addition, mRNA expression was determined for various mitochondrial solute and ion transporters. Intriguingly, levels of *SLC25A26*, a nuclear gene encoding the S-adenosylmethionine carrier (SAMC) localized in the mitochondrial inner membrane, were increased 2.7 ± 0.5-fold (*p* < 0.05) in myopathy patient fibroblasts compared to healthy control fibroblasts (Figure 7A). Gene expression levels of mitochondrial ion transporters, including *VDAC1,2,3, SLC25A4,* and *SLC25A6*, were not significantly changed, though all appeared elevated in the myopathy-derived skin fibroblasts (Figure 7B–G and Figure S2). Levels of *SLC25A5*, encoding the mitochondrial ADP/ATP translocase 2 (ANT2), were close to significantly increased in myopathy compared to healthy controls (*p* = 0.056; Figure 7F). Moreover, expression of *PLSCR3,* encoding phospholipid scramblase 3, which is associated with mitochondria-mediated apoptosis, was not significantly changed in myopathy skin fibroblasts compared to control skin fibroblasts (Figure 7H).

### 2.6. No Overall Difference in DNA Methylation as Detected by LC-MS/MS

**Figure 8:**
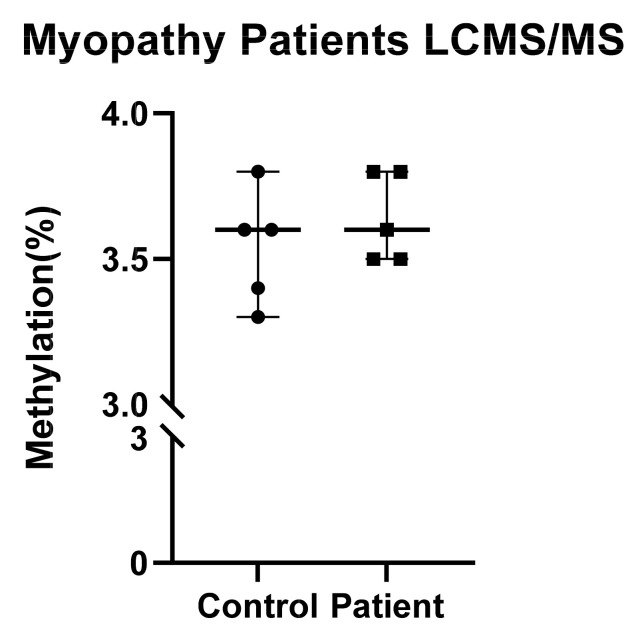
LC-MS/MS analysis of patient and control fibroblast samples, measured in 2–4 independent passages (see Table S4 for reproducibility). (Control samples are shown as dots, and patient samples as squares).

The increase in *SLC25A26*, encoding the S-adenosylmethionine carrier SAMC, tempted us to assess total mtDNA methylation levels. LC-MS/MS analysis was first optimized to robustly determine the percentage of methylated C in commercial fibroblasts HDFn16 cells (Figures S3 and S4). Next, five patient and five control fibroblasts were analyzed, resulting in reproducible methylation percentages of approximately 3.5% (Figure 8, Table S4), without differences between patients and controls. Despite the effective yield of mtDNA, nDNA was detectable using our procedure (Figures S5 and S6). To reduce the amount of nDNA, Trizol RNA phases were assessed for mtDNA yield and nDNA contamination. For well-known cell lines (HEK293, HepG2), as well as for skin fibroblast cultures, the nDNA contamination was lower in Trizol RNA fractions compared to Abcam isolates, with similar amounts of mtDNA yield. The improved mtDNA purity was reflected by a lower percentage of mC in Trizol RNA fractions compared to our Abcam isolates (Figures S5 and S6), requiring further optimization of mtDNA isolation procedures for LC-MS/MS.

## 3. Material and Methods

### 3.1. Patient Samples

The study protocol was reviewed and approved by the local ethics committee of the University Medical Center of Groningen, Groningen, the Netherlands (UMCG METc 2017.444).

### 3.2. Muscle Biopsy and ATP Measurement

After local anesthesia and aseptic cleaning of the skin, a 5 mm diameter needle was inserted into the muscle of the upper leg to remove a small sample of muscle. Fresh muscle biopsies, obtained from 14 myopathy patients and 4 healthy individuals, were partly snap frozen in liquid nitrogen and partly directly placed in SETH medium containing 0.25 mol/L sucrose, 2.0 mmol/L potassium-EDTA, 10.0 mmol/L Tris and 5.104 U/L heparin (fresh) to determine ATP-generating capacity [33,34]. The myopathy patients and control individuals were all of pediatric age.

### 3.3. Skin Biopsy and Fibroblast Culture

For five of the 14 myopathy patients, we could obtain skin biopsies to be subcultured as fibroblast cell lines. Five fibroblast cell lines obtained from nonmyopathy individuals were included as controls, together with the commercial human dermal fibroblast (Gibco™ C0045C) lines HDFa16 and HDFn16. Fibroblast cell lines were cultured in Ham’s F10 + l-Glutamine medium (Thermo Scientific, Waltham, MA, USA) containing 5% fetal bovine serum (Lonza, Basel, Switzerland) and 1% penicillin/streptomycin/fungizone (Lonza) at 37 °C in a humidified atmosphere containing 5% CO_2_. Cells were trypsinized at 80–90% confluency and cultured with fresh medium in a 25 cm^2^ culture flask at 10^5^ cells in 5 mL at seeding. Subculture took place in a 175 cm^2^ culture flask. At 80–90% confluency, cells were harvested and washed twice in ice-cold PBS. The cells were pelleted at 800× *g* at 4 °C, and stored at −80 °C for further analysis.

### 3.4. Human Skeletal Cell Line Culture and Treatment with S-Adenosyl Methionine (SAM)

Human skeletal muscle cells (HSkMC) (Cell Applications, Inc., San Diego, CA, USA), were grown in HSkMC growth medium (Cell Applications) at 37 °C in a humidified atmosphere containing 5% CO_2_. At 80–90% confluency, cells were trypsinized and subcultured with fresh medium in a 25 cm^2^ culture flask at 10^5^ cells in 5 mL at seeding. The numbers of cells for the experiments were determined using the TC20™ automated cell counter (Bio-Rad, Hercules, CA, USA). Ultimately, cells were subcultured in 175 cm^2^ culture flasks at a confluency of 80–90%.

HSkMCs were subjected to S-adenosylmethionine (SAM) (Sigma-Aldrich, St. Louis, MO, USA) in different concentrations varying between 50 µmol/L and 1000 µmol/L. The SAM was dissolved in water, sterilized through a 0.45 µm filter, and further diluted with HSkMC medium (Cell Applications). Cells were harvested after 2 days.

### 3.5. MtDNA, Total DNA, and RNA Isolations

MtDNA was isolated from frozen muscle tissue crushed with liquid nitrogen in a tissue grinder and from frozen fibroblast cell pellets using a Mitochondrial DNA Isolation Kit (Abcam #65321, Cambridge, UK) according to the manufacturer’s instructions. Alternatively, total DNA and RNA were extracted from frozen skin fibroblasts and HSkMCs using the AllPrep DNA/RNA/Protein Mini Kit (Qiagen, Hilden, Germany) according to the manufacturer’s protocol. The DNA and RNA concentrations in each sample were measured with a NanoDrop spectrophotometer (Thermo Scientific). The input for LCMS/MS was based on readings obtained using the Qubit™ dsDNA HS and BR Assay kits (Thermo Scientific).

### 3.6. Pyrosequencing

**Figure 9:**
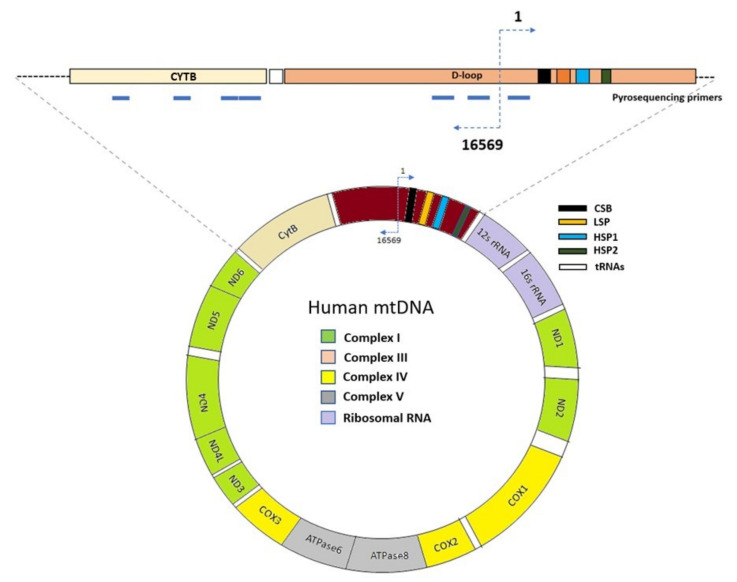
Annotated representation of human mitochondrial DNA, including regions analyzed by pyrosequencing in the current study. These include regions within the *CYTB* gene (14698–15761, 15798–15812) and *D-loop* region (163–187, 16084–16457).

Prior to pyrosequencing, Fast Digest *Bam*HI (Thermo Scientific) treatment was conducted to linearize the mtDNA at 37 °C for 1 h. There is only one *Bam*HI restriction site in mtDNA (14258–14263). Total DNA or mtDNA samples (500 ng) were bisulfite-converted using an EZ DNA Methylation-Gold Kit (Zymo Research, Irvine, CA, USA) according to the manufacturer’s protocol. Bisulfite-converted DNA (20–50 ng) was subjected to PCR of mitochondrial *D-loop* regions and *CYTB* gene using the Pyrosequencing PCR Kit (Qiagen). Primers were designed using the PyroMark Assay Design 2.0 software (Qiagen), and a BLAST search (https://blast.ncbi.nlm.nih.gov/Blast, 1 May 2017) was carried out to exclude primers recognizing nuclear mitochondrial DNA sequences (NUMTs). Primer locations are shown in Figure 9 and Table S1. Prior to pyrosequencing, the PCR products were validated for correct size using 2% agarose gel electrophoresis. Cytosine methylation was quantified by pyrosequencing using sequencing primers (Table S1). The methylation percentage at each cytosine in any context (CpN) site was quantitatively analyzed using the PyroMark Q48 Autoprep Software (Qiagen) according to the manufacturer’s instruction.

### 3.7. Real-Time PCR (qRT-PCR)

RNA expression of different genes in skin fibroblasts and human skeletal muscle was analyzed by quantitative real-time PCR (qRT-PCR) as described previously [22]. Total RNA was treated with DNase I (Thermo Scientific). RNA was reverse transcribed using random hexamer primers with M-MLV reverse transcriptase to generate cDNA according to the manufacturer’s protocol (Thermo Scientific). For the mitochondrial genes (*ND1,* NADH dehydrogenase subunit 6 (*ND6), CYTB,* and cytochrome c oxidase subunit 1 (*COX1)*), each qRT-PCR reaction contained 10 µmol/L of the antisense and sense primers (Table S2) (Sigma-Aldrich), 10 ng cDNA, and 2x Absolute QPCR SYBR Green Rox Mix (Thermo Scientific). For nuclear genes encoding mitochondrial transporters, TaqMan gene

Supplementary Materials: expression assays were performed for *SLC25A4* (hs00154037_m1), *SLC25A5* (hs00854499_ g1), *SLC25A6* (hs00745067_s1), *SLC25A26* (hs01115565_m1), voltage-dependent anion channel 3 (*VDAC3)* (hs01091534_g1), voltage-dependent anion channel 2 (*VDAC2)* (hs00762994_s1), voltage-dependent anion channel 1 (*VDAC1)* (hs040978484_m1), and phospholipid scramblase 3 (*PLSCR3)* (hs02339687_ g1) (Thermo Scientific), containing 20× Taqman Gene Expression assay, 10 ng cDNA, and 2× Taqman Universal PCR Master Mix on the ViiA7 Real-Time PCR System (Thermo Scientific). Β-actin was used as the reference gene for nuclear and mitochondrial genes. Relative expression compared to controls was calculated using the ΔΔCt method [35].

### 3.8. Mitochondrial DNA Copy Number Measurement (q-PCR)

Human mtDNA copy number was determined by qPCR using primers designed for *CYTB*, *β-actin,* and GAPDH by calculating the ratio of the nDNA average CT value to the mtDNA average CT value (Table S2). A total of 30 ng of total DNA was analyzed per sample. Real-time qPCR was carried out on the ViiA7 Real-Time PCR System (Thermo Scientific) for 15 min at 95 °C, followed by 40 cycles of 15 s at 95 °C, 30 s at 60 °C, and 30 s at 72 °C.

### 3.9. Mass Spectrometry

The acetonitrile, ammonium acetate, and acetic acid were of analytical grade (Merck, Darmstadt, Germany). The 2′deoxycytidine, 2′deoxycytidine-15N3, 5-methyl-2′deoxycytidine, 5-methyl-2′deoxycytidine-d3, 5-(hydroxy)methyl-2′deoxycytidine, 5-(hydroxy)methyl-2′deoxycytidine-d3, thymidine, and thymidine (13C10,15N2) were purchased from Cambridge Isotope Laboratories (Tewksbury, MA, USA). Individual standards were prepared by dissolving to 1 mg/mL in distilled water. A working standard mixture of 100 µg/mL was prepared by diluting the intermediate stock standard solution, from which the calibration standards within the range of 10–100,000 ng/mL were prepared by serial dilution with acetonitrile/water (9:1, *v*/*v*). The solutions included Solution A (0.8% acetic acid and 10 mmol/L ammonium acetate in aqueous solution) and B (0.1% acetic acid in acetonitrile) [36].

The mtDNA was digested (Nucleoside Digestion Mix, cat.no. M0649S, New England Biolabs, Ipswich, UK), and 5 µL was subjected to HILIC-U(H)PLC–SCIEX API 4500QQQ analysis according to Zhou et al. [36], with minor adaptations (Shimadzu LC- 30AB U(H)PLC coupled with a SCIEX API 4500QQQ instead of the Waters system). U(H)PLC linear gradient conditions were: 0–6 min, 10% A–90% B; 6–9 min, 40% A–60% B; 9–11 min; 50% A–50% B. Mass spectrometry detection was performed by using a SCIEX API 4500QQQ (AB Sciex, Framingham, MA) equipped with an electrospray ionization (ESI) source operating in positive ionization mode. The desolvation gas flow rate was set to 1000 L/h at a temperature of 550 °C, the cone gas flow rate was set at 50 L/h, and the source temperature was set at 150 °C. The capillary voltage was set to 3000 V; the cone voltage was dependent upon the MRM for each compound. Data were collected in multiple-reaction monitoring (MRM) mode by screening precursor and product ions simultaneously. MRM transitions for the compounds are shown in Supplement Table S3. For data acquisition, Analyst version 1.6.2 was used. Methylation percentage was determined as the ratio of (5mC/ (5mC + dC))×100.

### 3.10. Statistical Analysis

Statistical analysis was performed using GraphPad Prism 7 software (San Diego, CA, USA). Differences between patients and healthy samples or between tissue groups were determined using the Mann–Whitney U-test. Data are presented as individual symbols or as box plots showing minimum and maximum values (ends of the whiskers), interquartile range (length of the box), and median (line through the box), as well as outliers. Correlation analysis was conducted using Pearson’s correlation test. In all analyses, a *p*-value of 0.05 or less was considered statistically significant (* *p* ≤ 0.05).

## 4. Discussion

In this study, we explored whether analyzing the mitochondrial genome beyond its genetic sequence would provide biomarkers for myopathy patients who did not harbor known disease-associated genetic mutations. We here provide indications that mtDNA methylation in the *CYTB* gene was elevated in muscle tissue of 14 myopathy patients when compared to healthy controls, and that this parameter correlated with ATP production. In this pilot study, we focused on methylation changes in a *CYTB* region, because *CYTB* mutations associate with mitochondrial disorders and encephalomyopathy [11,37]. In addition, cytosines in the *D-loop* were analyzed due to their importance in regulating mtDNA transcription and replication. Indeed, for various diseases, differential *D-loop* methylation has been described [23,38,39]. Although no differences between myopathy and healthy muscle samples were found for the *D-loop* regions, when considering all analyzed cytosines, the average mtDNA methylation correlated with the ATP-generating capacity. This finding warranted further analysis of mtDNA to pinpoint regions of importance for myopathies, as well as the overall mtDNA methylation status, using bisulfite-independent techniques.

Our finding that mtDNA methylation in muscle was associated with mitochondrial dysfunction in myopathy was in line with findings in other diseases [21,23,38,40,41,42]. For example, differential methylation has been associated with disease state in steatohepatitis patients [16]. In some studies, differential mtDNA methylation correlated with decreased expression of mtDNA-encoded genes [16,23,38]. Unfortunately, only limited sample volume can be obtained through muscle biopsies, so we could not address mtDNA gene expression in the same muscle samples. In future studies, we will explore mtDNA of easy-to-obtain cells isolated from urine, which mimic the metabolism of muscle biopsies quite well [43]. Although noninvasive, again, limited cell numbers are obtained from urine. The current study therefore included skin fibroblasts, obtained through low-invasive approaches and subcultured in vitro. Per individual pediatric myopathy patient, there was a good correlation between muscle and fibroblast CpN methylation. However, the myopathy-associated increase in *CYTB* methylation in muscle was not convincingly mimicked in fibroblasts. Interestingly, it has been reported that the methylation status of mtDNA in human fibroblasts decreases with culture age, and that this was more pronounced in fibroblasts obtained from young donors compared to fibroblasts of old donors [44]. Our tight correlation in mtDNA methylation between muscle and fibroblasts, however, justifies further study to assess whether skin biopsies can replace the more invasive discomforting procedure of muscle biopsies [45,46].

Skin fibroblasts obtained from myopathy patients did show a trend toward an increase in mtDNA methylation in *CYTB* at CpT position 15803. However, this increase did not result in changes in *CYTB* gene expression in the cultured skin fibroblasts. As mtDNA copy numbers, in accordance with higher *PPARGC1A* expression, tended to be higher in patient-derived fibroblasts, the mtDNA methylation might act to prevent an increased expression of particular mitochondrial genes. This would explain the lack of differential mtDNA gene expression in myopathy fibroblasts compared to healthy samples. Indeed, there is evidence in some mitochondrial diseases that mtDNA over-replication acts as a compensatory mechanism to mask the effects of mtDNA mutations [20,45,47]. In muscle-specific TFAM knockout mice, used to model myopathy, increased mitochondrial copy numbers were found as potential compensation mechanism for the reduced function of the ETC [48]. In addition, in amyotrophic lateral sclerosis (ALS) patients, an increase in mitochondrial copy number was observed, although this was associated with a decrease in *D-loop* methylation [39].

The enhanced *SLC25A26* expression observed for myopathy skin fibroblasts does indicate an increase in mitochondrial SAM uptake compared to healthy fibroblasts. Although overexpression of the transporter in a cervical cancer cell line promoted non-CpG mtDNA hypermethylation [17], we did not detect a higher overall mtDNA methylation in myopathy skin fibroblasts compared to controls. Interestingly, SAM did dose-dependently increase mtDNA methylation in our HSkMCs. This increase was accompanied by enhanced expression of mtDNA-encoded genes and of *PPARGC1A,* as well as mtDNA copy number. Similarly, mitochondrial density increased in skeletal muscle in diabetic mice subjected to SAM treatment [49]. Although these observations may be explained by SAM-induced nuclear changes, increased methylation level of *PPARGC1A* was negatively correlated with *PPARGC1A* mRNA and with mtDNA content in muscle of diabetic subjects [50]. Our previous studies on artificially induced mtDNA methylation demonstrated a direct causal effect in mtDNA methylation leading to reduced mtDNA gene expression [22]. Altogether, it seems that mtDNA expression is not only dependent on copy number, but can also be suppressed by methylation in a cell-type- and CpN-dependent manner.

Interestingly, like compaction of nDNA in heterochromatin, DNA supercoiling is widely investigated and known to influence transcription of genes in bacterial genomes [51]. Since mtDNA forms a supercoiled structure, just like bacterial plasmid DNA, it is likely that supercoiling has a regulatory function in mtDNA gene expression and replication as well. TFAM covers the entire mitochondrial genome, and can induce U-turn bends in the promoter-containing *D-loop* regions, thereby helping to organize mtDNA [52]. Importantly, TFAM binding is affected by mtDNA methylation [53,54], providing a mechanistic link between mtDNA methylation and gene expression regulation. The supercoiled mtDNA structure, as well as the extensive coverage by proteins [29], might render some cytosines more resistant to bisulfite conversion required for pyrosequencing, prompting researchers to linearization/sonication [25,29,55] and/or additional protein removal before conversion [28]. Ignoring this bisulfite resistance might have resulted in overestimation of mtDNA methylation levels, and in the persistent controversy on whether mtDNA methylation actually exists [26,55]. Together with suboptimal mtDNA sample preparation, standard technical readouts often disregard strand specificity and methylation in the non-CpG context [28]. It is important to note that techniques that are independent of bisulfite conversion, such as LC-MSMS, have confirmed mtDNA methylation [27,28]. We here first observed an overall methylation percentage of about 3.5%, but we could not fully rule out the potential effects of nuclear DNA contamination. In fact, reducing nDNA contamination by analyzing mtDNA in the Trizol RNA phase resulted in a lowering of 5mC% as measured by LC-MS/MS. These and other [27,29] findings require more detailed investigations before conclusions on the presence of mtDNA methylation can be made. Indeed, a recent mass spectrometry study convincingly demonstrated the presence of adenosine methylation in mtDNA [56]. Although detected at very low levels (0.04%), the mtDNA adenosine methylation changes were functionally associated with mtDNA transcription differences. For future studies, we propose to analyze mtDNA fragments to enrich them for potentially methylated sites, while at the same time removing nDNA contamination [57].

The bisulfite-dependent pyrosequencing data indicating methylation differences between muscle and fibroblasts and between patient and control samples might thus represent bisulfite inaccessibility more than (or in addition to) DNA methylation. These inaccessibility patterns, analogous to heterochromatin configuration of nuclear DNA, can be related to, for example, differential TFAM binding [54]. Such mitochondrial epigenetic mechanisms provide exciting alternative avenues to explore the many diseases associated with mitochondrial dysfunction. In line with an induction of mitochondrial biogenesis in myopathy patients, mtDNA epigenomic mechanisms may have a regulatory function that (a) impairs mitochondrial energy metabolism, leading to the development of myopathy; or (b) compensates for mitochondrial biogenesis in response to the low ATP-generating capacity (Figure S7).

Unraveling such parameters of the mitochondrial genome beyond its sequence might present novel opportunities to interfere with mitochondrial dysfunction in myopathies. Indeed, gene-targeting platforms allow the selective deletion of mutated mitochondrial genomes [58,59]. These platforms are routinely repurposed to actively interfere with nuclear gene expression levels [60]. Similarly, DNA-targeting platforms can be exploited to interfere with mitochondrial DNA methylation levels [53], resulting in gene expression changes [22]. So, in addition to providing molecular biomarkers, our findings open novel avenues to clinically interfere with (unexplained) myopathies.

## Data Availability

The datasets analyzed during the current study are available from the corresponding author on reasonable request.

## Supplementary Materials

The following supporting information can be downloaded at: https://www.mdpi.com/article/10.3390/ijms23042197/s1.
