# Supplementary material for: The Mitochondrial Epigenome: An Unexplored Avenue to Explain Unexplained Myopathies?
Source: Int J Mol Sci. 2022 Feb 16;23(4):2197. doi: 10.3390/ijms23042197 (PMC8879787; doi:10.3390/ijms23042197)
Supplement: Supplementary file 1 [file ijms-23-02197-s001.zip › ijms-1558440-supplementary.pdf]

Supplementary Figures

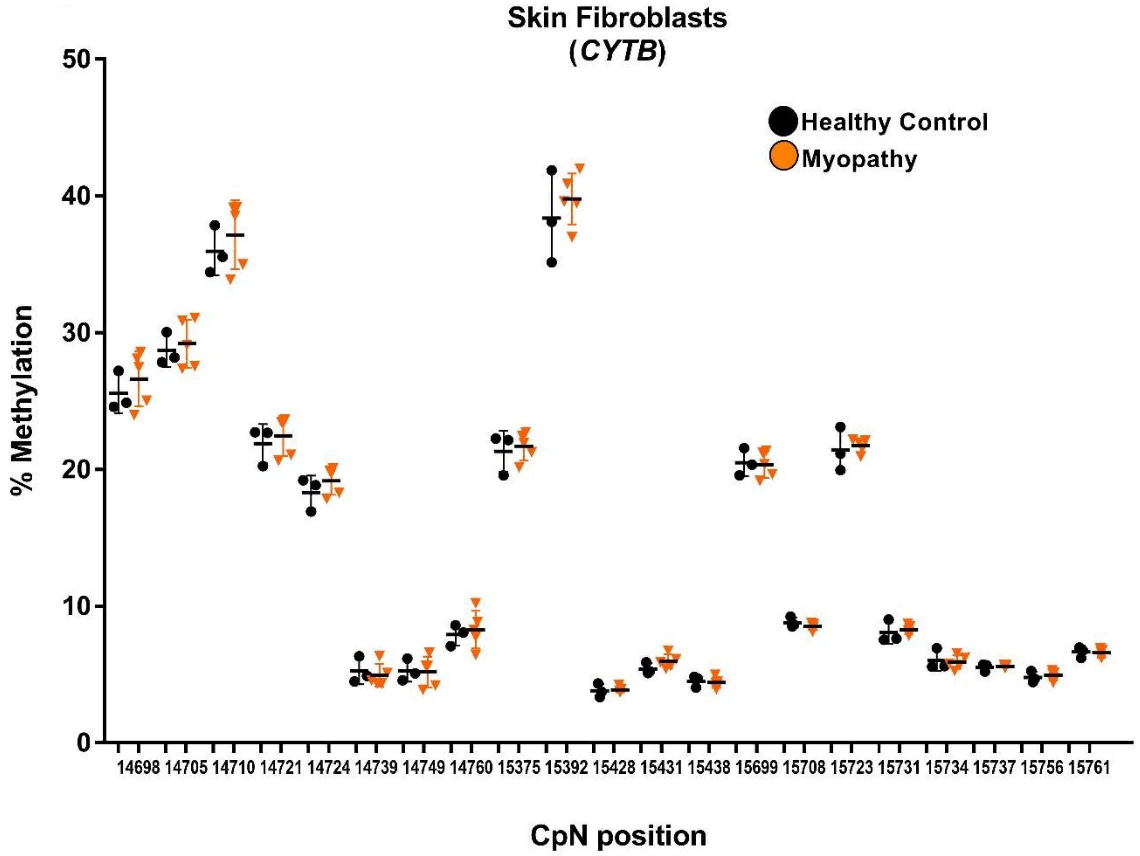

Figure S1. Extended analysis of *CYTB* methylation in skin fibroblasts from myopathy patients compared to healthy samples. MtDNA pyrosequencing analysis of skin fibroblasts from healthy controls and myopathy patients for *CYTB* gene (14698 – 15761) (healthy controls, n = 3 and myopathy patients, n = 5).

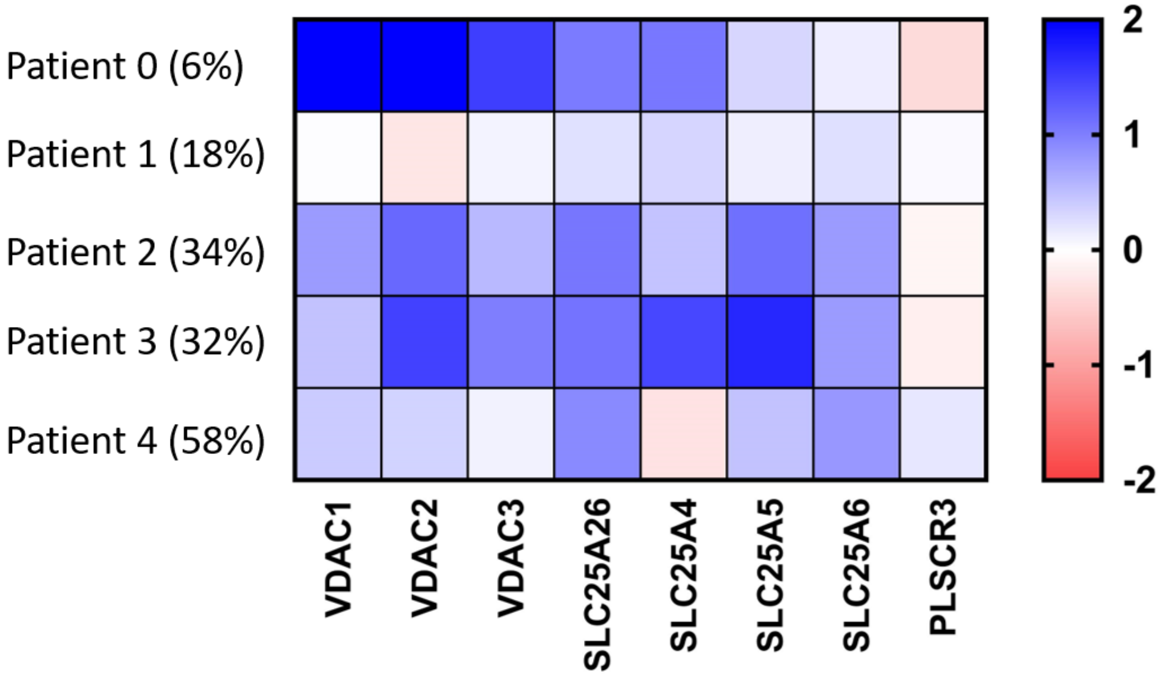

Figure S2. Heatmap showing normalized gene expression of mitochondrial transporters (*SLC25A4-6, SLC25A26*, *VDAC1-3* and *PLSCR3*) in skin fibroblasts from myopathy patient related to those from healthy controls. The ATP-generating capacity in the corresponding muscle tissues ranged from 6 to 58%. Skin fibroblasts were obtained from myopathy patients (Pt0, Pt1, Pt2, Pt3 and Pt4), with ATP-generating capacity of 6%, 18%, 32%, 34% and 58%, respectively.

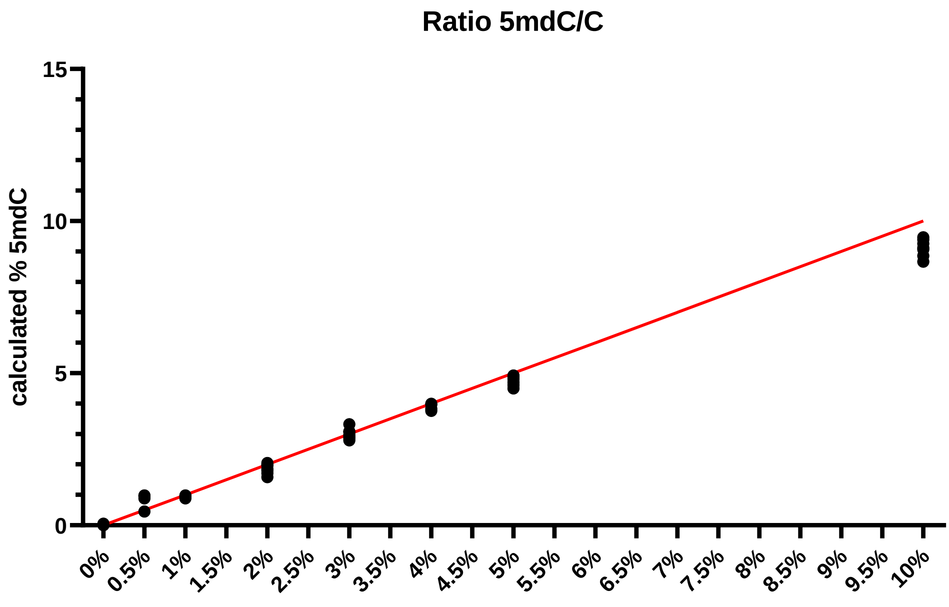

Figure S3. Linearity of %5mC**.** Three independent mixtures with varying amounts of mC and C (together making up 25%) plus equal amounts of T, G and A (together 75%) nucleosides were measured in three separate runs, each in triplicate. The solid red line indicates the calculated values.

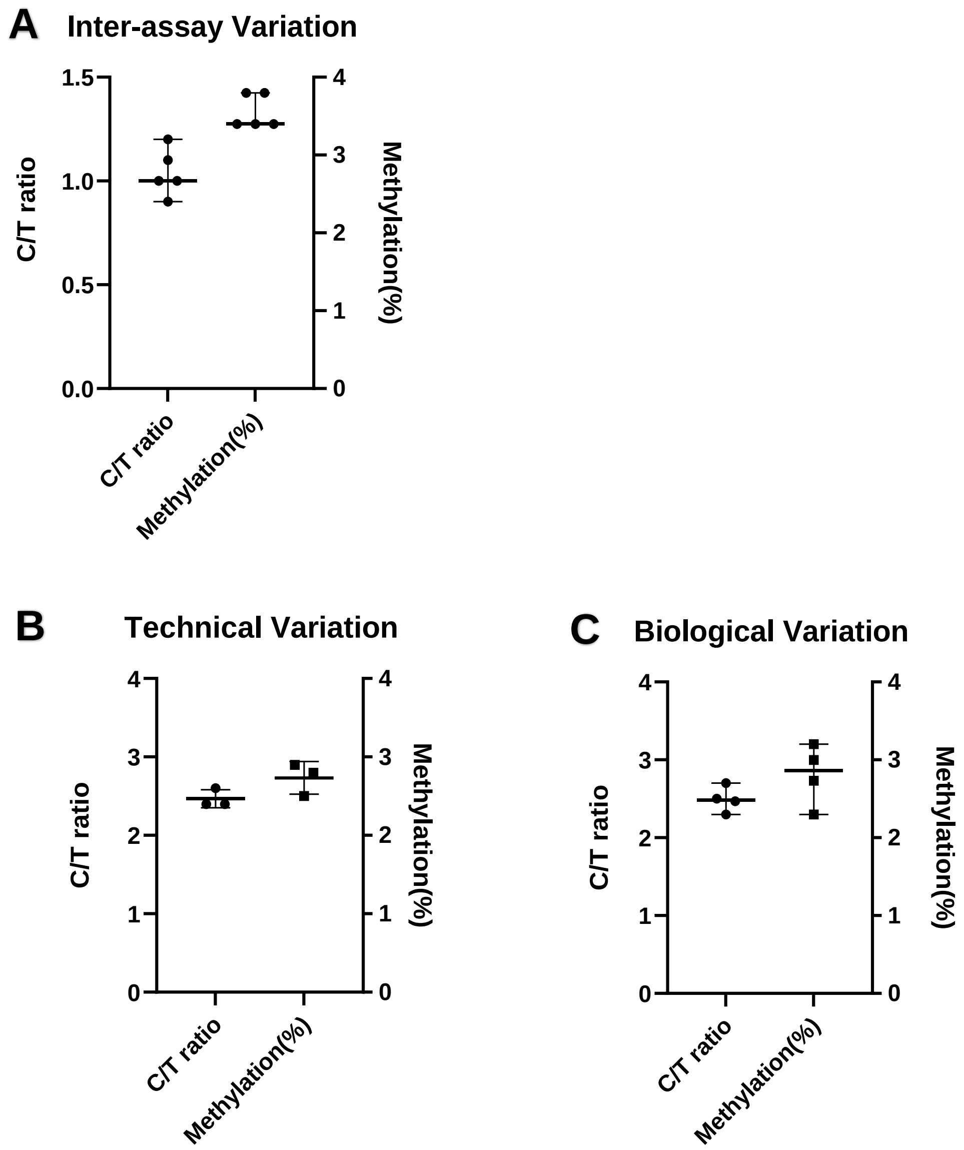

Figure S4. Reproducibility of mass spectrometry measurements of cytosine methylation and C/T ratio determined using commercially available skin fibroblasts HDFn16s (Gibco) isolated using Abcam kit. A) inter-assay variation: the same DNA isolate was measured at five different runs, B) technical variation: DNA isolates of three different pellets of one passage (P24), and C) biological variation: DNA isolates of cell pellets of four different passages (P18,20,24,25) were measured in one run.

LC-MS/MS:
Trizol RNA= 2.0,3.1 and 2.1, 2.2%
Abcam= 2.9 and 2.9, 2.7%

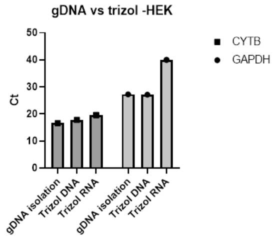

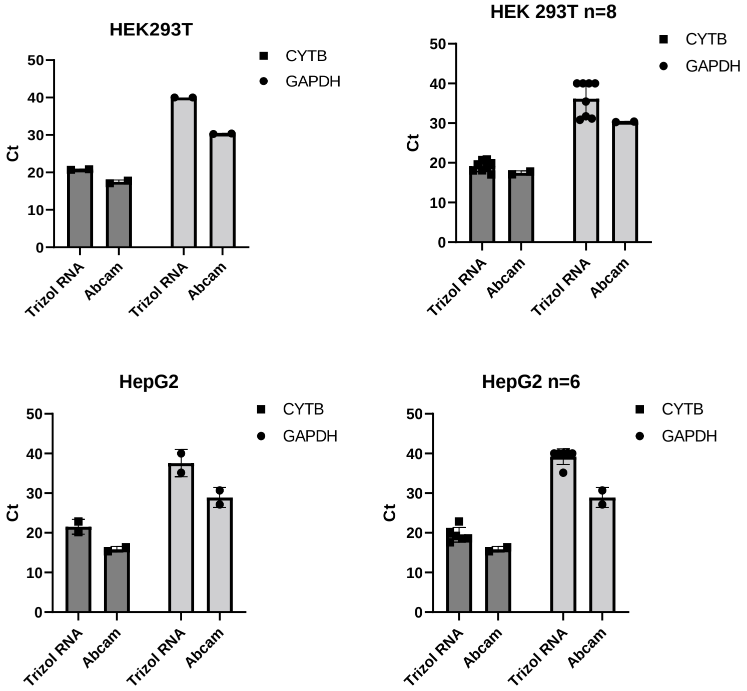

Figure S5. Contamination of nDNA in Abcam mtDNA isolates and enrichment of mtDNA in RNA phase of Trizol. Pellets of the same passage (n=2; left panels) or various passages (n=6-8; right panels) (of HEK293T, top; HepG2, bottom) were isolated using standard Trizol or Abcam procedures and 1ng (Nanodrop) was analyzed by qPCR to detect mtDNA (*CYTB*, dark bars) or nuclear DNA (GAPDH, light bars). Values are depicted as Ct values: the higher the value, the lower the amount of DNA.

For the two HEK293 pellets of left panel, LC-MS/MS was performed as described in M&M: the percentages of mC (analysed in duplicate by LC-MS/MS on two different days) are depicted at the top left. For one additional sample (of the n=8, right panel), also gDNA and DNA phase of Trizol isolation were included (see insert top right).

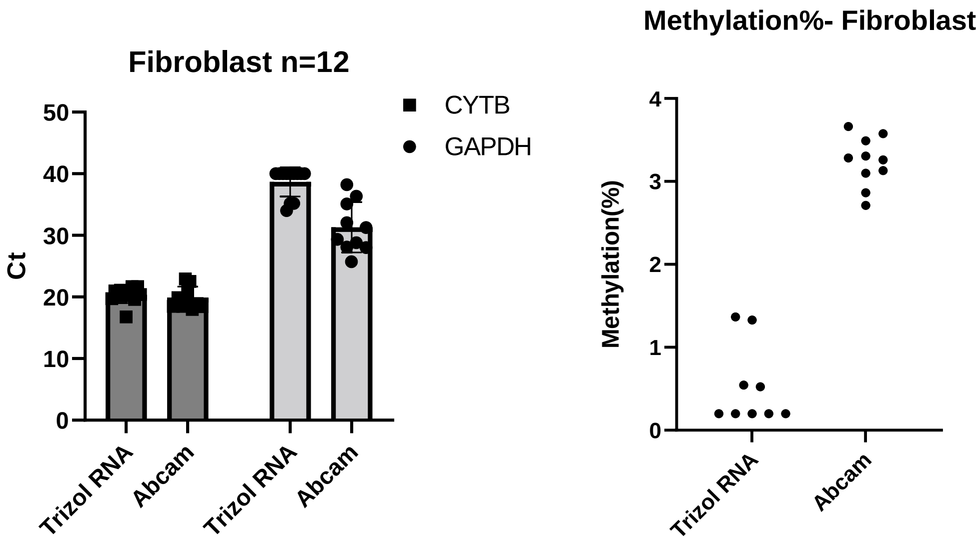

Figure S6. Enrichment of mtDNA in RNA phase of Trizol of skin fibroblasts. MtDNA of fibroblast culture pellets was isolated using the indicated method (RNAphase of Trizol or Abcam kit) and analyzed for mtDNA yield (*CYTB*) and nDNA contamination (GAPDH) (left panel). Comparison for %mC by LC-MS/MS of the different isolates is shown in the right panel, with five out of the nine samples below detection limit (represented at 0.2%).

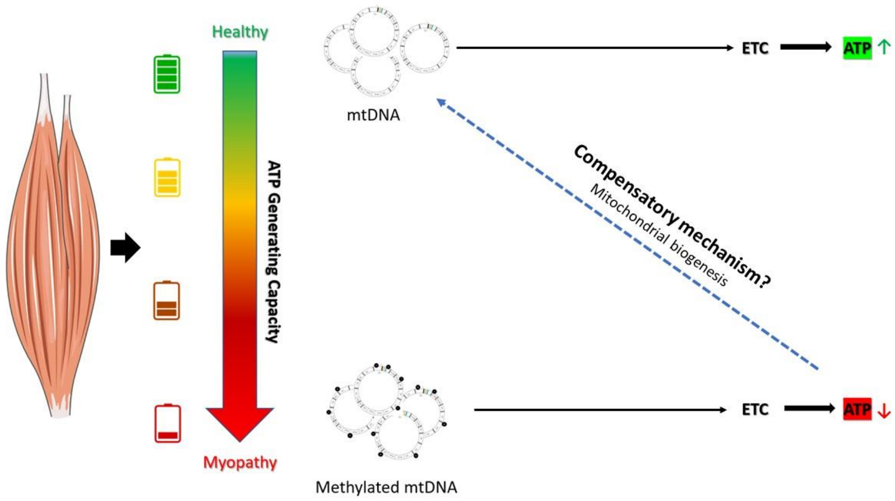

Figure S7. Diagram illustrating the proposed compensatory mechanism initiated in myopathy to restore the impaired ATP-generating capacity in muscle tissue. We hypothesize that mtDNA epigenome parameters impair the ETC, which in turn reduces

ATP generation. An increase in mtDNA copy number is an indicator of mitochondrial biogenesis, as the system tries to restore ATP generation to normal levels.

Supplementary TABLES

Table S1. Pyrosequencing primers for human mtDNA.

Region	Target Location	Primer sequence
D-loop	16412 - 16457	Fw: GGGTTATTTAGGTTTTATGATTTTGAAG<br>Rv: ATAACACATTACAATCAAATCCCTTCTC<br>Seq: GTTTATTTTAGTTATTTTTAAGTGT
	16084 - 16131	Fw: GGTTGATTGTTGTATTTGTTTGTAAGT<br>Rv: CACCATTAACACCCAAAACTAAAATTCTA<br>Seq: TTTATGTATTATAGGTGGTTAAG
	163 - 187	Fw: GTTTGGTGGAAATTTTTTGTTATGATGT<br>Rv: CTTTAATTCCTACCTCATCCTATTATTT<br>Seq: AATTAATATATTTTAGTAAGTATG
CYTB	15756 - 15812	Fw: TTAATTAGGGAGATAGTTGGTATTAGGA<br>Rv: CAATAATCCCCATCCTCCATATATCC<br>Seq: AGGATTGTTGTGAAGT
	14698 - 14760	Fw: GGGAGGTAGATGAATGAGTGGTTAAT<br>Rv: CAAACCCCATTACTAAACCCACACTC<br>Seq: TGGTTAATTAATTTTATTAGGGG
	15375 - 15438	Fw: GAGGTTTGGTGAGAATAGTGTT<br>Rv: CTTTACCTTTCACTTCATCTTACC<br>Seq: AAGGAGAGAAGGAAGA
	15699 - 15761	Fw: GTTTAATGATGGTAAAAGGGTAGTT<br>Rv: TAACAATAATCCCCATCCTCCATATATC<br>Seq: GGGTAGTTTATTGGTTGTT

Table S2. qRT-PCR primers for human mtDNA RNA expression. For copy number, CytB primers were used on non-reverse transcribed isolates. Values were normalized versus nDNA as determined by primers for genomic B-actin (qPCR).

Primer	Target	Primer sequences
GAPDH(qPCR)	Internal reference (housekeeping)	Fw: CCCTTCATACCCTCACGTATTC<br>Rv: CCATTCTGTCTTCCACTCACTC
B-actin(qPCR)	Internal reference (housekeeping)	Fw: TGAGTGGCCCGCTACCTCTT<br>Rv: CGGCAGAAGAGAGAACCAGTGA
B-actin(qRT-PCR)	Internal reference (housekeeping)	Fw: CCACCGCGAGAAGATGA<br>Rv: CCAGAGGCGTACAGGGATAG
ND1	A subunit of complex I	Fw: ATACCCCCGATTCCGCTACGAC<br>Rv: GTTTGAGGGGGAATGCTGGAGA
ND6	A subunit of complex I	Fw: GGGTGGTGGTTGTGGTAAAC<br>Rv: CCCGAGCAATCTCAATTAC
CYTB	A subunit of complex III	Fw: AATTCTCCGATCCGTCCCTA<br>Rv: GGAGGATGGGGATTATTGCT
COX 1	A subunit of complex IV	Fw: CGATGCATACACCACATGAA<br>Rv: AGCGAAGGCTTCTCAAATCA
PPARGC1A	A transcription coactivator in the regulation in energy metabolism and mitochondiral biogenesis	Fw: TGAGAGGGCCAAGCAAAG<br>Rv: ATAAATCACACGGCGCTCTT
TFAM	Mitochondrial transcription factor and has an effect on mtDNA replication	Fw: CCGAGGTGGTTTTCATCTGT<br>RV: TCCGCCCTATAACGATCTTG
NRF-1	A transcription factor and regulates mtDNA transcription and replication	Fw: GGGAGCTACAGTCACTATGG<br>Rv: TCCAGTAAGTGCTCCGAC

Table S3. LC-MS/MS multiple-reaction monitoring (MRM) transition for the tested compounds.

Q1=precursor ion; Q3= product ion.

	Q1 mass (Da)<br>(precursor ion)	Q3 mass (Da)<br>(product ion)
2’Deoxycytidine,	227.946	112.020
2’Deoxycytidine-15N3	231.100	115.000
5-methyl-2’Deoxycytidine	241.980	126.000
5-methyl-2’Deoxycytidine-d3	244.984	129.000
5-(hydroxy)methyl-2’Deoxycytidine	257.962	142.000
5-(hydroxy)methyl-2’Deoxycytidine-d3	260.978	145.000

Thymidine	242.900	127.000
Thymidine (13C10,15N2)	254.900	134.000

Table S4. Reproducibility of methylation percentages as measured by LC-MS/MS for independent fibroblast cultures (I-IV) of five non-myopathy controls and five myopathy patients (Abcam isolates).

	Methylation level (%)				
**ATP generating capacity**	I	II	III	IV	Mean %
Control	3.4	3.5	3.7	3.5	3.4
Control	3.6	3.7	4.3	3.1	3.6
Control	3.2	3.4	4.2	-	3.6
Control	3.2	-	-	3.3	3.3
Control	3.4	4.1	4.0	-	3.8
Pt0 (6%)	3.6	3.8	3.7	3.5	3.6
Pt1 (18%)	3.4	3.8	3.8	3.8	3.5
Pt2 (32%)	3.6	3.7	4.0	-	3.8
Pt3 (34%)	3.9	-	-	3.7	3.8
Pt4 (58%)	3.0	3.6	3.8	-	3.5
